# Global research trends on the links between the gut microbiota and diabetes between 2001 and 2021: A bibliometrics and visualized study

**DOI:** 10.3389/fmicb.2022.1011050

**Published:** 2022-09-29

**Authors:** Boxun Zhang, Zishan Jin, Tiangang Zhai, Qiyou Ding, Haoyu Yang, Jia Wang, Lili Zhang, Linhua Zhao

**Affiliations:** ^1^Institute of Metabolic Diseases, Guang’anmen Hospital, China Academy of Chinese Medical Sciences, Beijing, China; ^2^Post-Doctoral Research Center, China Academy of Chinese Medical Sciences, Beijing, China; ^3^Graduate College, Beijing University of Chinese Medicine, Beijing, China; ^4^General Department, Guang’anmen Hospital, China Academy of Chinese Medical Sciences, Beijing, China

**Keywords:** gut microbiota, diabetes, research trend, bibliometrics, visualization

## Abstract

**Background:**

Over the past 20 years, evidence has suggested that gut microbiota plays an important role in metabolic homeostasis. The relationship between gut microbiota and diabetes has become the focus of considerable scientific interest. With the sharp increase in publications in this area, it is imperative to analyze the relevant articles using bibliometrics methods.

**Methods:**

Publications on “the gut microbiota and diabetes” were retrieved and downloaded from the Web of Science Core Collection database. Microsoft Excel 2020, VOSviewer, CiteSpace 5.8.R3 and Co-Occurrence 9.94 software were used for data analysis and visualization. Country/academic institution, journal, author, subject category, keyword and reference were analyzed thoroughly. The cutting-edge directions in this field were also determined by analyzing keywords and key articles.

**Results:**

A total of 2,342 documents were included in the analysis; the number of articles in this field has increased yearly, particularly after 2010. China and the University of Copenhagen are the country and research institution associated with the largest number of publications. *Nutrients* have published 191 articles in this field, ranking first among highly productive journals in the number of publications. The researcher *Cani PD* affiliated with the University of Leuven, Belgium, published the greatest number of articles in this field between 2001 and 2021 and was also ranked as the first co-cited author and the largest contributor of highly cited papers in this field. *Endocrinology & Metabolism* was the most common subject category. Three of the most frequently found keywords, besides terms related to “microbiota” and “diabetes,” were “obesity,” “probiotics,” and “inflammation.” *Akkermansia muciniphila*, *Faecalibacterium prausnitzii*, trimethylamine n-oxide and branched-chain amino acids are intestinal bacteria or metabolites that have attracted more attention in recent years. Natural products represented by Chinese herbal medicine and some protein receptors or signaling pathways such as aryl hydrocarbon receptor, farnesoid X receptor and AMP-activated protein kinase were frontiers in this field.

**Conclusion:**

Over the past two decades, the rapid development of research on the gut microbiota has deepened the understanding of the physiology and pathology of diabetes, providing new insights into different approaches to treatment. In the future, further interdisciplinary innovation, clinical transformation, and application may receive more attention.

## Introduction

Over the past 20 years, significant progress has been made in intestinal microecology, and the relationship between the gut microbiota and multiple inflammation-related diseases has gradually become a research hotspot (Boulangé et al., 2016). Accumulating evidence suggests that diabetes, primarily type 2 diabetes (T2D), is a chronic systemic inflammatory disease (Hotamisligil, 2006), and compared with normal subjects, the intestinal flora of patients with T2D was characterized by a decrease in butyrate-producing bacteria and an increase in various opportunistic pathogens (Qin et al., 2012). Similarly, intestinal flora disturbance is also found in patients with type 1 diabetes (T1D), mainly characterized by differences in *Bacteroides spp.*, *Streptococcus spp.*, *Clostridium spp.*, and *Bifidobacterium spp.* (Jamshidi et al., 2019), and for infants, the α-diversity of the gut microbiota showed a remarkable downward trend before the diagnosis of T1D (Kostic et al., 2015). In addition, the main metabolites/products of the gut microbiota, such as short-chain fatty acids (SCFAs), lipopolysaccharides (LPS) and bile acids (BAs), also play important roles in regulating the metabolic homeostasis of the host (Dehghan et al., 2014; Zhao et al., 2019). Compared with the healthy group, the concentration of SCFA in the feces and circulation of patients with diabetes decreased, whereas the content of LPS increased significantly; these changes can promote damage to the intestinal barrier and low-grade systemic inflammation, subsequently inducing insulin resistance (IR) (Saad et al., 2016).

Diabetes treatment strategies and methods targeting the gut microbiota have also attracted wide attention. Results from a meta-analysis suggested that supplementation with probiotics, prebiotics, or synbiotics could improve metabolic outcomes in patients with diabetes (Bock et al., 2021). Some polyphenols, polysaccharides, or other active substances extracted from functional foods or herbs can also regulate glucose metabolism by modulating gut microbiota (Lyu et al., 2017). The molecular mechanism of intestinal microecological agents in treating diabetes may involve multiple pathways such as anti-inflammatory, antioxidant, intestinal barrier protection, and intestinal hormone regulation (Kim et al., 2018).

In addition, interdisciplinary integration is a significant feature in this field, which further increases the complexity of the knowledge structure. Although there has been a considerable expansion in articles on “gut microbiota and diabetes,” to the best of our knowledge, there is no research analyzing the basic information presented in the publications and exploring the changing trends in research topics. Bibliometrics is a subject that applies mathematical and statistical methods to analyze the knowledge structure and development trends of publications (Pritchard, 1969). Data integration and clustering can identify the salient authors, journals, and academic institutions in this field as soon as possible and accurately screen out the frontier research (Agarwal et al., 2016). In recent years, various new methods have emerged in the field of bibliometrics, providing ideas for the in-depth development of related research, at the same time, a large number of bibliometrics articles are also published in an increasing trend year by year (He et al., 2017; Zyoud et al., 2019; Yu and Pan, 2021). This study intends to apply bibliometrics to analyze the relevant information of the articles on “the gut microbiota and diabetes” published between January 2001 and December 2021 to improve understanding of the research history and status of current knowledge in this field, straighten out the publication trend, and explore the research highlights.

## Materials and methods

### Sources of data and search strategy

In order to ensure the authority of the original documents, data were retrieved and downloaded from the Web of Science Core Collection (WoSCC) (indexes: Science Citation Index Expanded [SCI-E]). To further examine the latest trends developing in this field, the time limit was from January 2001 to December 2021, which is also a period when major breakthroughs have been made in the research of gut microbiota. In order to facilitate the statistical analysis of literature data, we only included English documents. The scope of the retrieval was limited to Web of Science (WOS) database subject words, and the terms of the search strategy are shown in Table 1.

**TABLE 1:** Search strategy of Web of Science database.

Step	Search strategy
#1	TS = (gut OR intestin* OR gastrointestin*) AND TS = (microbio* OR microflora OR flora OR bacteri* OR dysbiosis OR microecology OR 16Sr* OR metagenome)
#2	TS = (prebiotic* OR probiotic* OR synbiotic*)
#3	#1 OR #2
#4	TS = (diabetes OR diabetic* OR IDDM OR NIDDM OR MODY OR T1D OR T1DM OR T2D OR T2DM)
#5	#3 AND #4

TS = Topic.

### Inclusion and exclusion criteria

Screening the retrieved literature is necessary to ensure the reliability of the data used for analysis. Two investigators (Boxun Zhang and Zishan Jin) independently reviewed the document according to the following criteria, and any differences were resolved through consultation with a third party.

**Inclusion criteria:** (1) the research topic of the article involves both the gut microbiota and diabetes and its related diseases (pre-diabetes, insulin resistance, diabetes complications); (2) the document type is “article” or “review articles”; (3) the document language is limited to “English”; and (4) the publication time is from 1 January 2001 to 31 December 2021.

**Exclusion criteria:** (1) the theme of the document is other metabolic diseases (such as obesity, non-alcoholic fatty liver, lipid metabolism disorder); (2) the topic of the study is not the gut microbiota but the urine, saliva or vaginal microbiota; (3) withdrawn or duplicate publications; and (4) documents that cannot provide the basic information required for bibliometric analysis.

### Data collection and analysis

The basic information in the records, such as article title, author, publication year, abstract, keywords, and citation frequency, were extracted and classified to analyze the data better. For some important articles, we searched the official websites of WOS and Scimago Journal & Country Rank (SJR) for their latest impact factors (IF), 5-year impact factor, quartile of a journal category and Hirsch index (H-index). The impact factor (IF) value was a quantitative index representing the influence of journals, which was determined based on the frequency of citations by other scientific publications (Garfield, 1999). The H-index, proposed by *J. E. Hirsch*, is another international evaluation index that can comprehensively quantify the academic contribution of scientists (Hirsch, 2005) and, at the same time, can be used to evaluate the influence of academic journals (Chen et al., 2020). Next, we used Microsoft Excel 2020 (Redmond, Washington, USA), VOSviewer (Leiden University, Leiden, the Netherlands), CiteSpace V 5.8.R3 (Drexel University, Philadelphia, PA, USA) and Co-Occurrence 9.94 (COOC 9.94) to perform data statistics and visual analysis. Specifically, Microsoft Excel was used for managing, screening and ranking documents; VOSviewer was used to create network visualization maps to analyze the collaborative relationships between countries/regions, institutions, journals, authors and keywords, as well as the co-citation network of journals and authors; CiteSpace was used to capture key information with strong bursts during a specific period, to help us identify and further discuss hot topics; COOC was used for making frequency statistics on countries, keywords, journals, and analyzing the changes of subject categories.

## Results

### General characteristics of the retrieved documents

**FIGURE 1:**
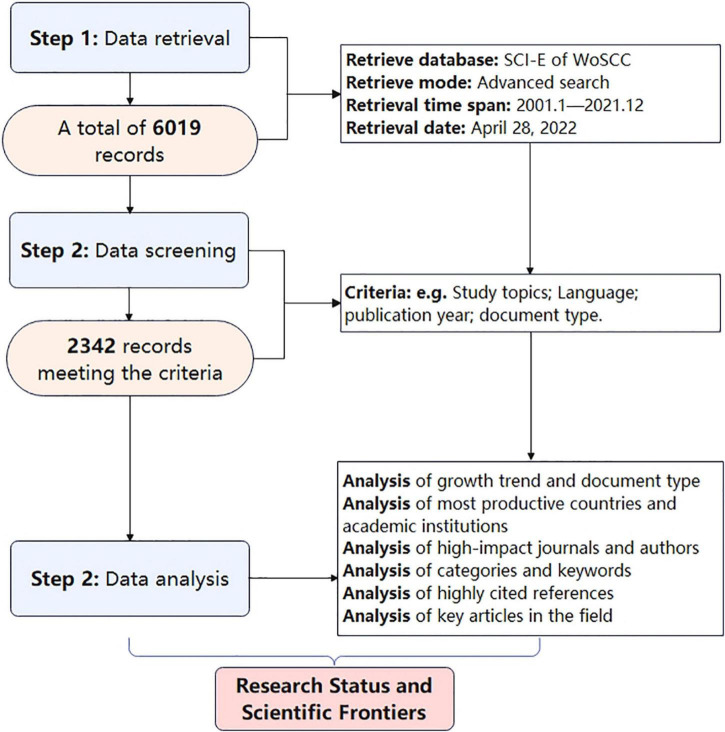
Flowchart of document search, screening, and data analysis.

According to the search strategy, a total of 6,019 documents were retrieved, but after screening according to the inclusion and exclusion criteria, only 2,342 documents could be used for further analysis (Figure 1). Of these, 73.5% were original, and 26.5% were review articles. In the past 21 years, the number of papers in this field has been increasing year by year, particularly after 2010.

### Country/region and academic institution distribution

**FIGURE 2:**
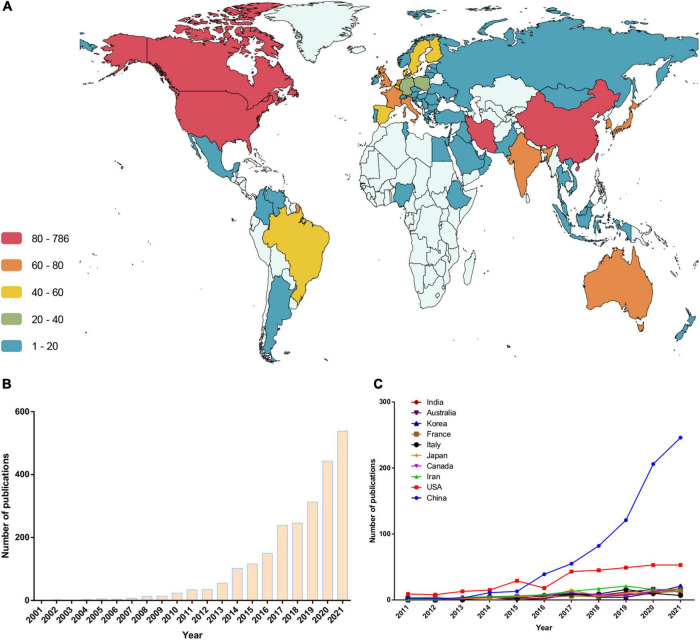
Trends in the number of publications and analysis of country/regions in the “gut microbiota and diabetes” area. **(A)** Geographical distribution of publication. **(B)** The trend in the total number of publications in the past 21 years. **(C)** The trend in the number of publications in the top 10 productive countries.

The authors of these articles were from 84 countries or regions. A corresponding author in China published a total of 786 articles, ranking first for the number of articles published. Authors ranking second to fifth were from the US, Iran, Canada, and Japan, respectively. Figure 2A shows the global distribution of research output in this field (based on the nationality of corresponding authors). In addition, we analyzed the change in trends in the annual number of publications from authors based in the countries above (Figure 2C and Table 2). Before 2015, the number of papers published by American scholars ranked first in the world, and over the past 5 years, papers from China increased sharply. However, regarding the citation frequency of each article, China still lags behind most countries (Table 2). In addition, several publications from other countries showed an increasing trend year by year (Figure 2B).

**TABLE 2:** Top 10 productive countries/regions related to the research field of the gut microbiota and diabetes.

Rank	Countries/ regions	Total number of papers	N/2342	Continents	Total citations	Citations per article
1	China	786	33.6%	Asia	20,076	25.5
2	USA	346	14.8%	North America	19,783	57.2
3	Iran	108	4.6%	Asia	3,639	33.7
4	Canada	81	3.5%	North America	4,187	51.7
5	Japan	74	3.2%	Asia	3,101	41.9
6	Italy	72	3.1%	Europe	3,609	50.1
7	France	67	2.9%	Europe	13,375	199.6
8	Korea	67	2.9%	Asia	2,806	41.9
9	Australia	65	2.8%	Oceania	2,102	32.3
10	India	65	2.8%	Asia	2,046	31.5

**FIGURE 3:**
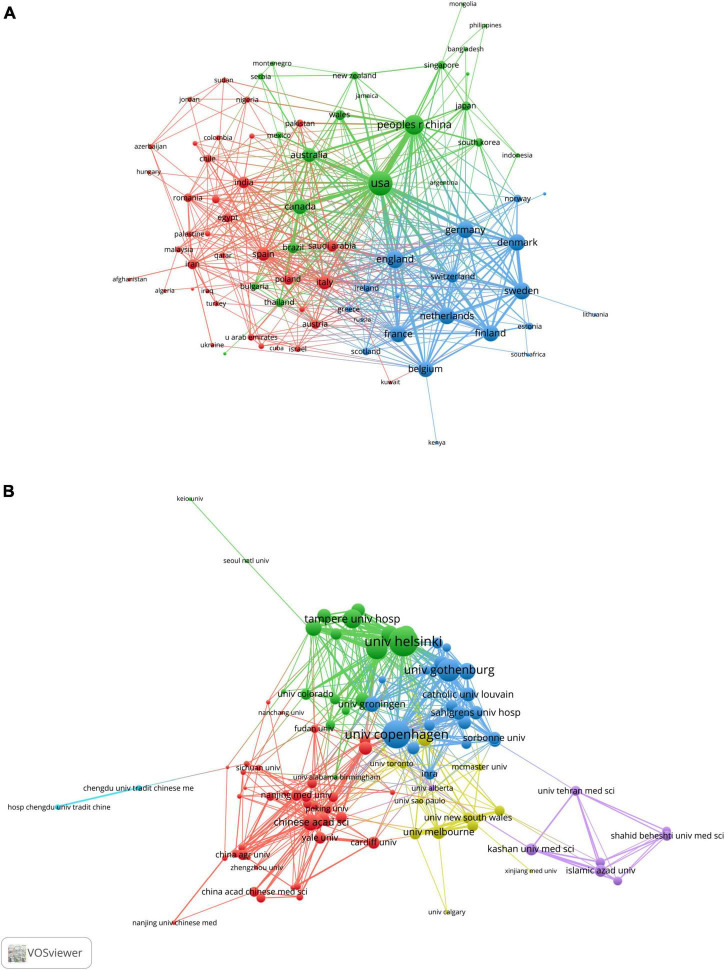
VOSviewer network visualization map of countries/regions and academic collaboration in the “gut microbiota and diabetes” area. **(A)** Collaboration network map of the most productive countries. **(B)** Collaboration network map of the most productive research institutions.

To further explore any cooperation relationship between countries/regions, we used VOSviewer software to perform a co-occurrence clustering analysis. The node’s size represented the strength of links to others, the thickness of the line represented the number of cooperation, and the same color meant that these countries or institutions had closer cooperation. As shown in Figure 3A, the USA had the highest total link strength, reflecting the closest level of cooperation with other countries, particularly China and Canada; some European countries/regions, such as England, France, Germany, Belgium, Sweden and Netherlands, jointly formed a blue cluster and also built close cooperation networks; additionally, Italy and Spain led the red clusters (Supplementary Table 1).

These studies included 2,670 academic institutions, with the University of Copenhagen, University of Helsinki, University of Gothenburg, Catholic University of Leuven and Chinese Academy of Sciences (CAS) ranking among the top five institutions for the number of papers published (Supplementary Table 2). Figure 3B shows the leading academic institutions in this field. Co-occurrence cluster analysis was used to explore the cooperative relationship among academic institutions. The results showed that the total link strength of the University of Copenhagen was the highest, indicating that it had the closest level of cooperation with other academic institutions. Furthermore, the University of Helsinki and the University of Gothenburg were key nodes of the collaboration network (Supplementary Table 2). Chinese research institutions represented by CAS, Shanghai Jiao Tong University and Zhejiang University formed the red group, suggesting extensive cooperation.

### Journal distribution

The 2,342 papers were published in 1,237 academic journals. Among the ten most productive journals, *PLOS One* was US-based, and the remaining were from Europe (Switzerland, UK, France and Germany). The average journal IF listed in Table 3 was 5.56 (IQR: 4.67–6.38), and their average H-index was 180.5 (IQR: 117.5–241.75). All the journals were classified as Q1 (the top 25% of the IF distribution) or Q2 (between the 50th and 25th percentile).

**TABLE 3:** Top 10 productive journals in the “gut microbiota and diabetes” area.

Rank	Journal	Country	Count (n)	IF (2021)	5-year IF	H-index	Quartile in category
1	Nutrients	Switzerland	191	6.706	7.185	143	Q1
2	Scientific Reports	UK	130	4.996	5.516	242	Q1
3	PLOS One	USA	110	3.752	4.069	367	Q2
4	Food & Function	UK	91	6.317	6.375	89	Q1/Q2
5	Journal of Functional Foods	UK	75	5.223	5.178	97	Q1/Q2
6	International Journal of Molecular Sciences	Switzerland	68	6.208	6.628	195	Q1/Q2
7	Biomedicine & Pharmacotherapy	France	61	7.419	6.581	109	Q1
8	Frontiers in Microbiology	Switzerland	50	6.064	6.843	166	Q1
9	Diabetes	USA	47	9.337	10.509	345	Q1
10	Diabetologia	Germany	46	10.46	10.617	241	Q1

**FIGURE 4:**
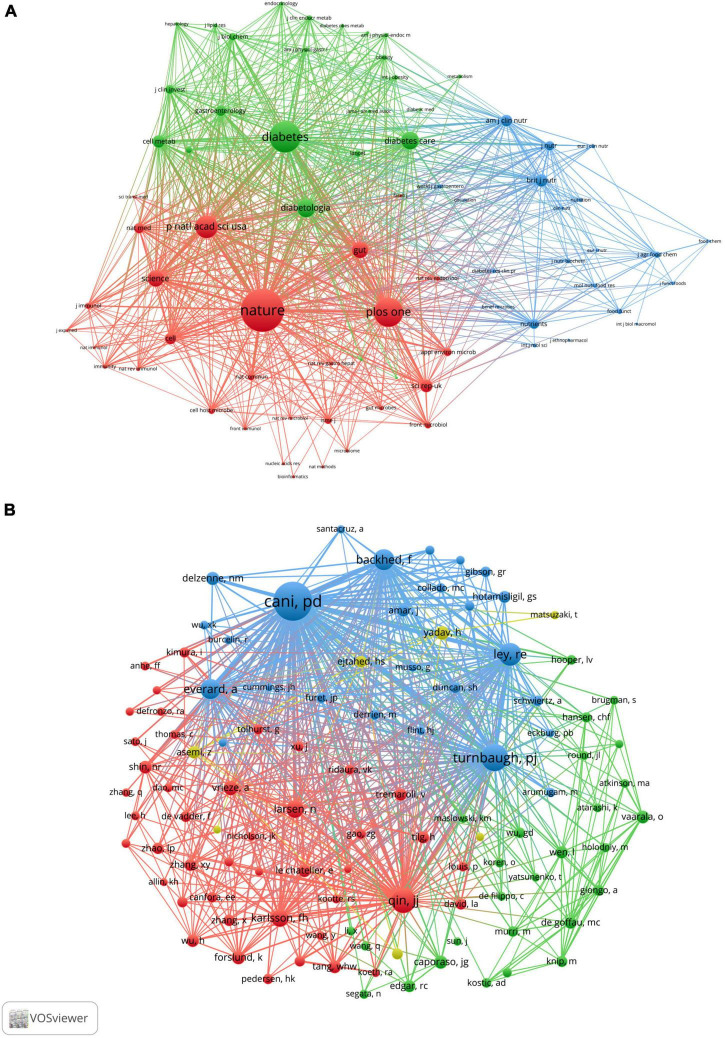
VOSviewer network visualization map of co-cited journals and authors of the publications related to the “gut microbiota and diabetes” area. **(A)** Visualization map of co-cited journals. **(B)** Visualization map of co-cited authors.

When two or more journals are cited by one article simultaneously, they form a co-citation relationship. Table 4 lists the top 10 co-citation journals, six from the USA, three from the UK and one from Germany. All journals except the *British Journal of Nutrition* and *PLOS One* were considered as Q1. We used VOSviewer software to perform a co-occurrence clustering analysis. The results showed that these co-citation publications could be divided into three clusters (Figure 4A and Supplementary Table 4): (1) comprehensive scientific journals in the red cluster, such as *Nature*, *PLOS One*, *PANS, Science*; (2) diabetes-related journals in green, such as *Diabetes, Diabetes Care, Diabetologia, Cell Metabolism*; and (3) food and nutrition-related journals in blue, such as *British Journal of Nutrition*, *American Journal of Clinical Nutrition*, *Nutrients*, *Journal of Nutrition*.

**TABLE 4:** Top 10 co-cited journals in the “gut microbiota and diabetes” area.

Rank	Journal	Country	Count (n)	IF (2020)	5-year IF	H-index	Quartile in category
1	Nature	UK	49.962	69.504	63.58	1,276	Q1
2	Diabetes	USA	3.24	9.337	10.509	345	Q1
3	PLOS One	USA	9.461	3.752	4.069	367	Q2
4	P. Natl. Acad. Sci. USA	USA	11.205	12.779	13.45	805	Q1
5	Diabetologia	Germany	47.728	10.46	10.617	241	Q1
6	Diabetes Care	USA	23.059	17.152	17.242	380	Q1
7	Gut	UK	19.112	31.793	27.827	311	Q1
8	Science	USA	7.045	63.714	59.924	1,229	Q1
9	British Journal of Nutrition	UK	10.122	4.125	4.862	198	Q3
10	Cell Metabolism	USA	22.682	31.373	35.104	292	Q1

### Author distribution

A total of 11,414 authors are included in these publications. Table 5 describes the top 10 most productive and co-cited authors’ basic information. It is important to note that nine of the most productive and six co-citation authors were from Europe. *Cani PD* of the *University of Leuven in Belgium* published the most publications in this field between 2001 and 2021, followed by *Nieuwdorp M*, *Delzenne NM*, *Bäckhed F*, and *Burcelin R*. If two or more authors appeared in the references of an article at the same time, they were considered as co-citation authors. These authors often had considerable research achievements and may be regarded as leading figures in the field. Table 4 lists the top 10 co-citation authors, and *Cani PD* and *Bäckhed F* also ranked among the top five in the high co-citation authors list, indicating that they published the greatest number of articles and had extensive international influence. Figure 4B shows the network visualization map of the co-cited authors. The node size represented the number of co-citations, and authors in the same color group were co-cited more frequently.

**TABLE 5:** Top 10 productive authors and co-cited authors in the “gut microbiota and diabetes” area.

Rank	Author	Count	Country	Rank	Author	Citations weight	Country
1	Cani PD	44	Belgium	1	Cani PD	2,070	Belgium
2	Nieuwdorp M	34	Netherlands	2	Turnbaugh PJ	1,035	USA
3	Delzenne NM	29	Belgium	3	Qin J	844	China
4	Bäckhed F	29	Sweden	4	Ley RE	734	USA
5	Burcelin R	26	France	5	Bäckhed F	630	Sweden
6	Asemi Z	24	Iran	6	Everard A	580	Belgium
7	Pedersen O	18	Denmark	7	Larsen N	447	Denmark
8	Wong FS	18	UK	8	Karlsson FH	446	Sweden
9	Hansen AK	16	Denmark	9	Vrieze A	307	Netherlands
10	Everard A	16	Belgium	10	Yadav H	274	USA

### Category analysis

The subject category represents the main research direction of a study. In general, 2,342 papers involved 88 WOS categories, and the top five subjects were identified as Endocrine & Metabolism, Nutrition & Dietetics, Food science & Technology, Biochemistry & Molecular biology, and Microbiology, accounting for 14.7, 10.9, 7.2, 6.8, and 6.7% of the total, respectively (Supplementary Table 4). Next, we paid more attention to the emerging categories in this field in the past 3 years. The weighted average year of occurrence of a specific category (with a frequency of at least five times) is calculated by using COOC software. The results show some categories not belonging to the biomedical category frequently appeared, such as Chemistry, Agriculture and Polymer Science. Clinical disciplines related to diabetes, such as Geriatrics & Gerontology, Urology & Nephrology, and Integrative & Complementary Medicine, started to increase after 2019 (Supplementary Table 5).

### Keywords analysis

**FIGURE 5:**
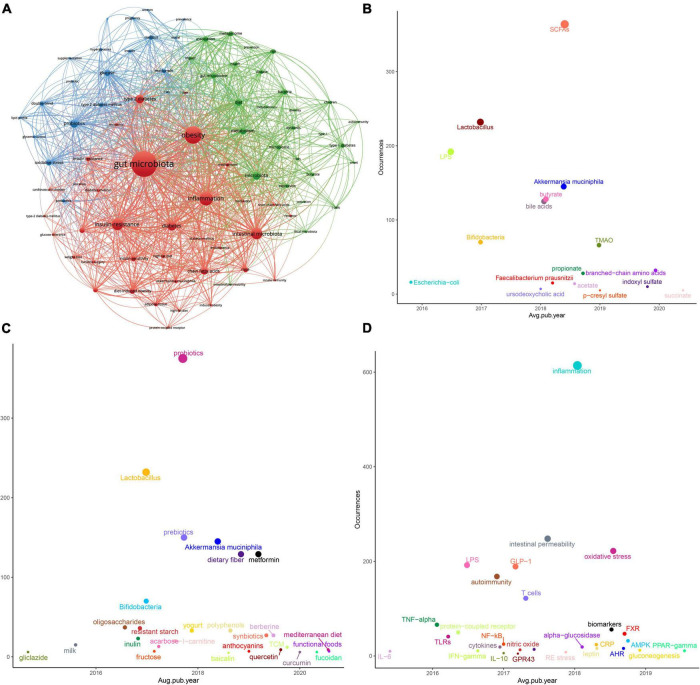
Keywords analysis in the “gut microbiota and diabetes” area. **(A)** Clustering analysis of high frequency keywords. **(B)** Coordinate diagram of frequency occurrence of the intestinal bacteria (and their metabolites). **(C)** Coordinate diagram of frequency occurrence of interventions. **(D)** Coordinate diagram of frequency occurrence of molecular mechanisms.

The 2,342 articles contained a total of 3,415 different keywords. It is noteworthy that several of the most frequently found keywords, besides terms related to “microbiota” and “diabetes,” were “obesity,” “probiotics,” and “inflammation.” To further understand the knowledge structure in this field, we performed co-occurrence analysis using VOSviewer software. As shown in Figure 5A, the size of the circle represented the total link strength, and the thickness of the line represented the number of co-occurrences. Finally, high-frequency keywords were clustered into three clusters. The red cluster showed some T2D-related keywords, such as obesity, inflammation, insulin resistance, and fatty acids. The green cluster mainly included T1D-related keywords, such as children, autoimmunity and nod mice. The red cluster mainly included keywords related to intestinal microecological agents, such as probiotics, prebiotics, and some words about clinical research, such as double-blind. The details are shown in Supplementary Table 6.

To better track research hotspots and frontiers, we applied the “overlay visualization” mode to analyze the keywords again. We classified the frequently occurring keywords according to three categories of the gut microbiota and its metabolites, intervention measures and molecular mechanisms. As Figures 5B–D shows, the abscissa represents the score calculated by the VOSviewer software based on the average year of publication, and the ordinate represents the weight of the keyword, that is, the frequency of occurrence. In recent years, in addition to some beneficial bacteria, such as “*Lactobacillus*,” “*Bifidobacteria*,” “*Akkermansia muciniphila* (*A. muciniphila*),” and “*Faecalibacterium prausnitzii* (*F. prausnitzii*),” metabolites or derivatives derived from the gut microbiota such as “SCFAs,” “LPS,” and “BAs” also received more attention. Still, after the year 2019, “trimethylamine n-oxide (TMAO),” “branched-chain amino acids (BCAAs),” “p-cresyl sulfate (PCS),” “indoxyl sulfate (IS),” and “succinate” gradually appeared in more scientific papers. Studies on natural products represented by Chinese herbal medicine (TCM) or their extracts have also appeared in large numbers. The keywords related to the mechanism were immune inflammation, protein receptor, glucose metabolism, and oxidative stress, among others. In the recent 3 years, “aryl hydrocarbon receptor (AhR),” “farnesoid X receptor (FXR),” “AMP-activated protein kinase (AMPK),” “gluconeogenesis,” and “Peroxisome proliferator-activated receptor-gamma (PPAR-γ)” were getting more attention.

### Reference analysis

When two or more publications were cited by one article simultaneously, they constituted a co-citation relationship. References with high co-citation are considered an important knowledge base in this field. We used VOSviewer software to screen out the ten most cited references and found they were published in *Nature* (four articles), *Diabetes* (two articles), *PANS* (three articles) and *PLOS One* (one article). Four articles focused on the relationship between obesity and gut microbiota. All these achievements were made under the guidance of *Gordon JI* of Washington University in the USA. Three articles (Larsen et al., 2010; Qin et al., 2012; Karlsson et al., 2013) reported the characteristics of intestinal flora in diabetic patients. Two articles (Cani et al., 2007a,b, 2008) discussed diabetes, intestinal flora and metabolic inflammation. One article explored the relationship between *A. muciniphila*, intestinal epithelium and diet-induced obesity. Table 6 provides further details.

**TABLE 6:** Top 10 co-cited references in the “gut microbiota and gut microbiota and diabetes” area.

Authors	Title	Article type	Year of publication	Source	Country of corresponding author
Qin et al	A metagenome-wide association study of gut microbiota in type 2 diabetes	Clinical research	2012	Nature	China
Turnbaugh	An obesity-associated gut microbiome with increased capacity for energy harvest	Animal experiment	2006	Nature	USA
Larsen et al	Gut microbiota in human adults with type 2 diabetes differs from non-diabetic adults	Clinical research	2010	PLOS One	Denmark
Cani et al	Metabolic endotoxemia initiates obesity and insulin resistance	Animal experiment	2007	Diabetes	France
Karlsson et al	Gut metagenome in European women with normal, impaired and diabetic glucose control	Clinical research	2013	Nature	Sweden
Cani et al	Changes in gut microbiota control metabolic endotoxemia-induced inflammation in high-fat diet-induced obesity and diabetes in mice	Animal experiment	2008	Diabetes	France
Ley et al	Microbial ecology: human gut microbes associated with obesity	Clinical research	2006	Nature	USA
Bäckhed et al	The gut microbiota as an environmental factor that regulates fat storage	Animal experiment	2004	PANS	USA
Ley et al	Obesity alters gut microbial ecology	Animal experiment	2005	PANS	USA
Everard et al	Cross-talk between Akkermansia muciniphila and intestinal epithelium controls diet-induced obesity	animal experiment	2013	PANS	Belgium

**FIGURE 6:**
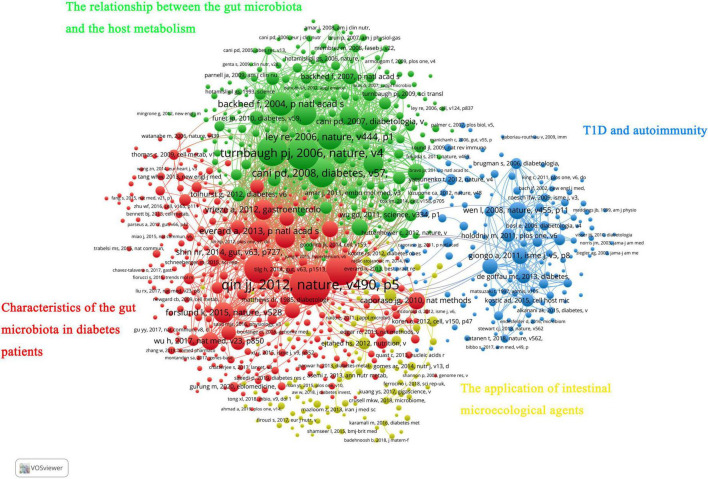
VOSviewer network visualization map of co-cited references.

In addition, all co-cited references were divided into four clusters. The red cluster mainly involved studies on the gut microbiota characteristics in the diabetes population and its influencing factors (such as metformin and flora transplantation). The green cluster was the knowledge base on the relationship between the gut microbiota and the host metabolism. Most of the studies in the blue cluster are about T1D and autoimmunity. Finally, the yellow cluster mainly included research papers on the application of intestinal microecological agents (Figure 6).

**FIGURE 7:**
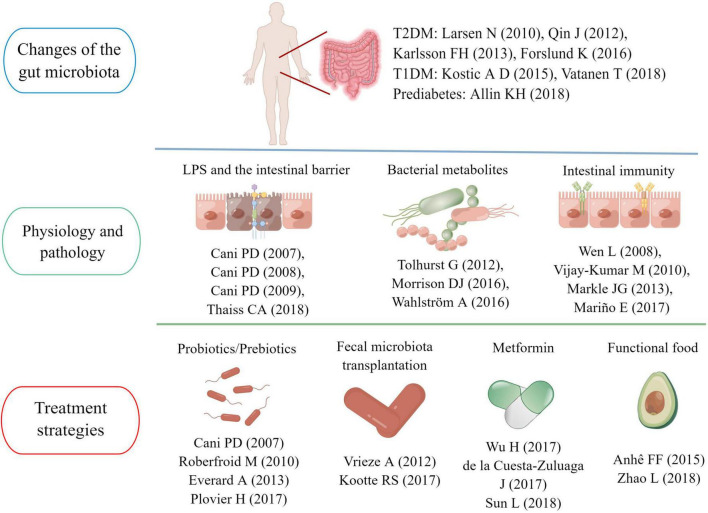
Main research topics of representative articles in the “gut microbiota and diabetes” area.

**FIGURE 8:**
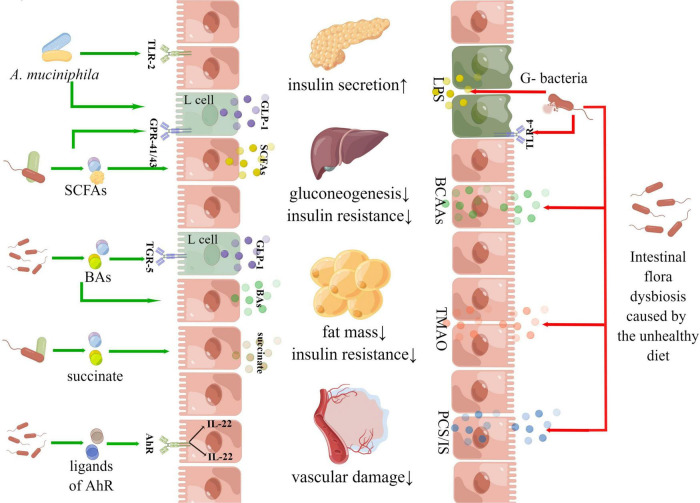
Diagram of hotspot mechanisms in the “gut microbiota and diabetes” area.

### Key articles in this field

We ranked the articles according to the number of citations (as of April 28, 2022). We listed the basic information (author, title, article type, year, country, and times cited) of the top 15 highly cited papers in Supplementary Table 7. These articles were published between 2007 and 2013 and consisted of nine animal experiments, four clinical studies, and two reviews. The articles were cited more than 1,000 times, and the highest number of citations was 3,474. In addition, according to the corresponding author’s country, 11 studies were conducted by research teams from European countries, the US research team mainly completed two studies, and the remaining two were from China and Canada.

Article citations are greatly affected by publication time. The citation times of articles published in recent 5 years are relatively low, even in important research articles. Therefore we used the “burst terms” function of CiteSpace to analyze the references with a sudden increase in citations in nearly 5 years. We have listed the basic information of the top 15 papers with the highest burst strength in Supplementary Table 8.

According to the above 30 landmark studies in this field, we sorted out the main research topics and development trends in the past 21 years. One of the hot research points is the gut microbiota characteristics of diabetic patients. Larsen et al. (2010) confirmed differences in the gut microbiota between healthy people and patients with T2D. Subsequently, researchers from China and Europe further identified the gut microbiota characteristics in individuals with T2D using metagenomic sequencing (Qin et al., 2012; Karlsson et al., 2013). A study published in 2015 pointed out that the use of metformin was a confounding factor that could not be ignored when analyzing the specificity of the gut microbiota in diabetic patients (Forslund et al., 2015). Nevertheless, metformin-induced gut microbiota modulation could benefit host metabolism, which has been confirmed in sterile mice (Wu et al., 2017). A study on community-dwelling Colombian adults found that diabetic patients taking metformin had a higher abundance of *A. muciniphila* and several gut microbiota known for producing SCFAs (de la Cuesta-Zuluaga et al., 2017). In recent years, research on gut microbiota characteristics has also been extended to people with pre-diabetes and T1D. The understanding of the relationship between the pathogenesis of diabetes and the gut microbiota is deepening (Kostic et al., 2015; Allin et al., 2018; Vatanen et al., 2018).

Functional interactions between the gut microbiota and host metabolism have been the primary research in this field in the past decade. The intestinal mucosal barrier is a research focus area (Tremaroli and Bäckhed, 2012). Cani et al. (2007a,2008) confirmed that intestinal barrier damage and excessive LPS entering the blood circulation were key links in the onset of diabetes and obesity, and regulating the gut microbiota could reverse the pathological process to a certain extent. Subsequently, the research team of Professor *Cani PD* further confirmed that the mechanism of improving the intestinal barrier by regulating the gut microbiota was related to increasing endogenous GLP-2 production (Cani et al., 2009). In 2018, a research team pointed out hyperglycemia could drive intestinal barrier permeability through GLUT2-dependent transcriptional reprogramming of intestinal epithelial cells and alteration of tight and adherence junction integrity (Thaiss et al., 2018).

Among the gut microbiota metabolites, SCFAs and BAs have attracted more attention. SCFAs are the products of indigestible carbohydrates fermented in the intestine, including acetic acid, propionic acid and butyric acid (Morrison and Preston, 2016). In 2012, *Tolhurst* and his colleagues confirmed that SCFA could trigger the secretion of GLP-1 in intestinal L cells through a G-protein coupled receptor-dependent pathway (Tolhurst et al., 2012). In 2018, a clinical study showed that fiber-rich diets could promote the production of intestinal SCFAs, increase GLP-1 secretion and improve hemoglobin A1c (HbA1c) levels in patients with T2D (Zhao et al., 2018). Another study found acetic acid and butyric acid could also prevent the occurrence of T1D by limiting the frequency of autoimmune T cells (Mariño et al., 2017). BAs are endogenous molecules synthesized by cholesterol in the liver and metabolized by the gut microbiota (Wahlström et al., 2016). Sun et al. confirmed metformin acted in part through a *Bacteroides fragilis –* the bile acid glycoursodeoxycholic acid (GUDCA) – intestinal farnesoid X receptor (FXR) axis to improve metabolic dysfunction, including hyperglycemia (Mariño et al., 2017). In addition, two review articles on intestinal flora metabolites also had a high number of citations, reaching 961 (Morrison and Preston, 2016) and 853 times (Sun et al., 2018), respectively.

The interaction between gut microbiota and the immune system has also aroused the interest of a large number of scholars. Wen et al. (2008) found germ-free MyD88-negative NOD mice would develop robust diabetes, but not the specific pathogen-free mice, which indicated the interaction of the gut microbiota with the innate immune system was a critical epigenetic factor modifying T1D predisposition. Besides, Markle et al. (2013) found that intestinal microflora could also prevent the occurrence of T1D by increasing serum testosterone levels (Vijay-Kumar et al., 2010). Intestinal immunity was also important for the occurrence of T2D. Vijay-Kumar et al. (2010) found that mice genetically deficient in Toll-like receptor 5 (TLR 5) tended to show the characteristics of multiple metabolic disorders, and these metabolic changes were correlated with changes in the composition of the gut microbiota (Markle et al., 2013).

Regulating the gut microbiota to achieve the goal of treating diabetes is also a hot topic in this field. Cani et al. (2007b) and Everard et al. (2013) found that *A. muciniphila* and selective increases of bifidobacteria could repair the damaged intestinal mucosal barrier, inhibiting systemic metabolic inflammation and improving insulin resistance. Subsequently, Plovier et al. (2017) confirmed that *Amuc_1100*, a specific protein isolated from the outer membrane of *A. muciniphila*, could play a therapeutic role in improving the intestinal mucosal barrier by interacting with toll-like receptor 2(TLR 2). Besides, various prebiotics and functional foods were also considered potential intestinal flora regulators (Roberfroid et al., 2010; Anhê et al., 2015), and research has shown blowout growth in the past decade. Fecal microbiota transplantation (FMT) is another feasible strategy. A clinical study published in 2012 found 6 weeks after infusion of microbiota from lean donors, insulin sensitivity of recipients increased along with levels of butyrate-producing intestinal microbiota (Vrieze et al., 2012). After that, another clinical study confirmed the metabolic improvement effects of FMT were closely related to the baseline fecal microbiota composition (Kootte et al., 2017).

## Discussion

### Research overview

In this study, we conducted a bibliometric analysis of publications with the theme of “gut microbiota and diabetes” between 2001 and 2021. We comprehensively described the distribution of countries/journals/authors/subjects in this field. We summarized the current research hotspots and developing trends by analyzing keywords and highly cited articles. Several published bibliometric articles have recently discussed the relationship between gut microbiota and other diseases such as obesity, irritable bowel syndrome, and brain diseases. This indicates that research progress in the field of gut microbiota is affecting the development of many disciplines (Ejtahed et al., 2019; Zyoud et al., 2019, 2021). In 2017, our team analyzed 100 highly cited articles on “the gut microbiota and diabetes” published between 2007 and 2015 and analyzed the distribution of article types, journals, countries, institutions and authors (Tian et al., 2017). In contrast, the current study included more important publications, particularly those published in the recent 5 years. Except for some basic bibliometric statistics, this study focused more on the distribution and changing trends in research topics over recent years so that readers can obtain a more comprehensive understanding of this research field.

The following reasons may explain why the gut microbiota has gradually become a hotspot in diabetes research. Firstly, the rise of systemic biology has changed the traditional research model. Compared with studying the function of a single molecule, scientists focus more on the interaction between different elements that affect life activities (Gu et al., 2020). With further research, the gut microbiota, previously regarded as a “forgotten organ,” has received renewed attention and is considered a key link in the system network (O’Hara and Shanahan, 2006). The strong demand for medical development has boosted the progress of related technologies. For example, the application of germ-free animals, microbial culture technology, high-throughput sequencing and multi-omics research methods not only enabled researchers to better analyze the composition and structure of the gut microbiota but also promoted the serial research on the functional interaction between the gut microbiota and the host (Allen-Vercoe, 2013; Grover and Kashyap, 2014; Whon et al., 2021). In addition, strong support from the government constitutes a solid foundation for the continuous promotion of the research. For example, the US National Institutes of Health (NIH) invested more than US $1 billion in human microbiome research between 2007 and 2016, including the Human Microbiome Project (HMP) program, with a total investment of US $215 million (NIH Human Microbiome Portfolio Analysis Team, 2019; Proctor, 2019). Similarly, some other countries have also set up research projects in microbiology, such as the Canadian Microbiome Initiative, the Japanese Human Metagenome consortium and the China Microbiome Project. These have extensively promoted the rapid development of microbiology and the cross-integration with other disciplines (NIH Human Microbiome Portfolio Analysis Team, 2019).

### Characteristics of publications

Regarding the national distribution of published publications over the past 21 years, the number of corresponding authors from China was the largest, showing a sharp upward trend after 2015. However, at the same time, it should be noted that the citation rate of Chinese papers was relatively low. On the other hand, France had the highest citation frequency among the top ten high-productive countries, which is directly related to the two highly cited papers written by *Burcelin R*. If these two articles are removed, the number of citations per French article decreases to 106.9. From the perspective of the research institutions involved in the article, the University of Copenhagen, the University of Helsinki in Finland, and the University of Gothenburg in Sweden were among the top five, and have established extensive contact with many international academic institutions, which reflected the fact that Nordic universities had a strong research tradition and global influence in this field. In addition, cluster analysis showed that European countries and European academic institutions have a close cooperation network, which may be related to the promotion of a series of research projects under the EU framework, such as the EU MetaHIT Project (the EU Project on metagenomics of the human intestinal tract) (Proctor, 2019).

### Development trend and research hotspots

Probiotics represented by *Lactobacillus*, *Bifidobacteria*, *A. muciniphila* and a variety of prebiotics were the most concerned intestinal flora regulators with the therapeutic effect on diabetes. In addition, the regulative action of some commonly used hypoglycemic drugs, such as metformin and acarbose, has also attracted more attention. But in recent years, more studies have focused on synbiotics, TCM and their extracts. As the new intestinal microecological agent, synbiotics are a mixture of probiotics and prebiotics. With the help of prebiotics, the possibility of probiotics settling in the intestine can be greatly increased, and their survival time can also be significantly prolonged (Markowiak and Śliżewska, 2017). TCM has been used in China for thousands of years. With further research, some extracts from the TCM, such as baicalin, berberine, quercetin, and curcumin, have proved to exert hypoglycemic effects by regulating the gut microbiota.

The results of co-cited references showed that the relationship between obesity and gut microbiota had laid the foundation for diabetes research. Professor *Gordon JI* from the Washington University School of Medicine made outstanding contributions to obesity research. Professor *Gordon JI*’s research team not only found that there excited a close relationship between host metabolic abnormalities and the gut microbiota but also applied some innovative experimental technologies such as germ-free mice to explore the causal link, which had a profound impact on the development of this field in the next decade (Bäckhed et al., 2004; Ley et al., 2005). With the deepening of research, more intestinal bacteria and metabolites closely related to diabetes have been identified. *A. muciniphila* colonization in the intestinal mucosa was a potential probiotic with metabolic regulating effects (Zhang et al., 2019). Its importance to the host included the uptake and utilization of nutrients, the protection of the intestinal barrier, and the maintenance of intestinal mucosal immune homeostasis (Macchione et al., 2019). Several studies have reported that the decrease in *A. muciniphila* was closely associated with the development of diabetes and obesity (Depommier et al., 2019). Oral administration of *A. muciniphila* could effectively improve glucose and lipid metabolism disorders in patients with metabolic syndrome (Depommier et al., 2019). *F. prausnitzii*, an important butyrate-producing bacteria, could play an anti-inflammatory and intestinal mucosal protection role by regulating intestinal immunity, which was essential to inhibiting the development of chronic systemic inflammation (Xu et al., 2020). TMAO was a metabolite of the gut microbiota closely linked to diabetes and cardiovascular diseases. It could inhibit insulin-related signal pathways, activate inflammatory reactions, promote hyperglycemia and decrease glucose tolerance (Li et al., 2022). Tang et al. (2017) found that the increase of plasma TMAO level in T2DM patients has a good predictive value for adverse cardiovascular and cerebrovascular events and death. BCAAs are a class of essential amino acids which mainly depend on food intake and are also regulated by the gut microbiota. Multiple clinical studies found the level of BCAAs in plasma of patients with diabetes increased significantly (Izundegui and Nayor, 2022), and the accumulation of BCAAs could inhibit the transport and utilization of pyruvic acid and fatty acid, promote glycogen synthesis and eventually lead to hyperglycemia (Cuomo et al., 2022). Succinate is a multifunctional metabolite produced by the interaction between the host and the gut microbiota, which could play a role similar to hormones and signal molecules by binding with the succinate receptor-1 (SUCNR1) (Canfora et al., 2019). De Vadder et al. (2016) found that succinate could activate intestinal gluconeogenesis, reduce the expression of hepatic glucose-6-phosphatase, and maintain blood glucose homeostasis by mediating “gut-liver” crosstalk. PCS and IS were two of the most well-studied uremic retention solutes and were formed from dietary amino acids by colonic bacteria that possess p-cresol- and indole-forming enzymes, respectively (Snelson et al., 2019). A meta-analysis confirmed that elevated levels of PCS and IS were associated with increased mortality in patients with chronic kidney disease. PCS was also related to an increased risk of cardiovascular events (Lin et al., 2015). With the in-depth study of diabetes kidney disease and diabetes-related cardiovascular risk, the attention on these two metabolites has gradually increased.

There were multiple pathways linking the gut microbiota and the host metabolism. In addition to the “intestinal barrier – metabolic inflammation,” “SCFA-GPR,” “BAs-FXR,” and autoimmunity have attracted much attention, AhR, AMPK and PPAR-γwere becoming research hotspots in recent years. The activation of aromatic hydrocarbon receptors could promote the secretion of IL-22 with intestinal mucosal protection and enhance the integrity of the intestinal mucosal barrier (Ning et al., 2019). A study found that mice with intestinal-specific AhR deficiency were more sensitive to DSS-induced intestinal inflammation and epithelial cell apoptosis than the control group, indicating that AhR plays an important role in maintaining intestinal barrier homeostasis (Krishnan et al., 2018). AMPK and PPAR-γboth were key molecular targets of host metabolism. AMPK was called an “energy receptor,” and once the ratio of AMP/ATP in cytoplasm increased or other factors activated AMPK, glucose utilization and fatty acid oxidation would be enhanced. Gluconeogenesis and lipid synthesis pathways will be inhibited to maintain the balance of cell energy metabolism (Foretz et al., 2019). PPAR-γplayed a significant role in regulating various biological processes such as lipid metabolism, lipogenesis, cell division and apoptosis. PPAR-γagonist (such as thiazolidinedione) could exert plenty of pharmacological effects contributing to metabolic regulation (Dali-Youcef et al., 2013). Figure 8 gives these details.

### Limitations and future research directions

In this study, we only searched the Web of Science database and only included the English literature, which inevitably caused the omission of the original literature.

In future studies, research on the following issues may become directions for further exploration. First, many studies still need to demonstrate the causal relationship between the intestinal flora and the host phenotype, which also relates to whether the therapeutic methods targeted to the intestinal flora can play ideal therapeutic effects. Second, more attention may shift from intestinal to extraintestinal and bacteria to other microorganisms. A complete set of mature technical methods has been formed in the field of intestinal bacteria, which to a certain extent, has stimulated the curiosity of researchers about the microbiome of other parts of the human body, such as tongue coating, urine, and skin. Besides bacteria, the relationship between viruses, fungi and glucose metabolism is also gradually being explored. Thirdly, disease prediction and individualized precision treatment based on the gut microbiota are the trends for future clinical application. However, there is still a lack of high-quality, evidence-based medical evidence, which needs to be repeatedly verified through large-scale clinical studies. Fourth, the standardized preparation process of intestinal microecological preparations and more convenient and efficient microbiota detection methods are the basis for further development of relevant industries. For clinicians, a standardized guideline on gut microbiota therapies may be the most urgent need.

## Conclusion

Research on the association between gut microbiota and diabetes has recently become a hot topic, and the number of articles is increasing yearly. Through bibliometric analysis, we concluded that the countries and institutions with the highest number of publications were China and the University of Copenhagen, respectively. The journal with the most publications is *Nutrients*; Professor *Cani PD* is the most productive scholar and an important contributor to highly cited papers, and *Endocrinology & Metabolism* is the most common subject category. The following keywords represented research frontiers: *A. muciniphila*, *F. prausnitzii* and metabolites of the intestinal flora TMAO and BACCs; natural products represented by the TCM; some metabolite receptors such as AhR, FXR, and signal pathways represented by AMPK and PPAR-γ. In future research, the clinical transformation of theoretical results and interdisciplinary innovation research may receive more attention.

## Data availability statement

The original contributions presented in this study are included in the article/Supplementary material, further inquiries can be directed to the corresponding authors.

## Author contributions

All authors listed have made a substantial, direct, and intellectual contribution to the work, and approved it for publication.
