# Supplementary material for: Global research trends on the links between the gut microbiota and diabetes between 2001 and 2021: A bibliometrics and visualized study
Source: Front Microbiol. 2022 Sep 29;13:1011050. doi: 10.3389/fmicb.2022.1011050 (PMC9557185; doi:10.3389/fmicb.2022.1011050)
Supplement: Supplementary file 1 [file Data_Sheet_1.docx]

Supplementary Material

**Supplementary Table 1. Top 30 countries with the total link strength in the co-occurrence cluster analysis of countries**

| label | cluster | weight<Links> | weight<Total link strength> | weight<Documents> | weight<Citations> |
| --- | --- | --- | --- | --- | --- |
| usa | 3 | 45 | 391 | 480 | 29122 |
| peoples r china | 3 | 32 | 226 | 786 | 22282 |
| england | 2 | 34 | 159 | 92 | 16123 |
| sweden | 2 | 21 | 150 | 93 | 12369 |
| denmark | 2 | 22 | 139 | 90 | 18835 |
| germany | 2 | 23 | 139 | 76 | 8456 |
| netherlands | 2 | 27 | 138 | 89 | 13397 |
| france | 2 | 28 | 135 | 103 | 24688 |
| finland | 2 | 19 | 128 | 83 | 13060 |
| belgium | 2 | 22 | 106 | 68 | 25984 |
| canada | 3 | 27 | 99 | 101 | 5388 |
| australia | 3 | 24 | 88 | 96 | 3173 |
| italy | 1 | 30 | 79 | 99 | 4734 |
| spain | 1 | 25 | 69 | 77 | 4623 |
| switzerland | 2 | 21 | 54 | 27 | 7183 |
| india | 1 | 24 | 51 | 74 | 2372 |
| saudi arabia | 1 | 21 | 45 | 23 | 2616 |
| brazil | 3 | 16 | 44 | 66 | 2969 |
| wales | 3 | 9 | 37 | 23 | 1545 |
| poland | 1 | 21 | 34 | 41 | 2027 |
| iran | 1 | 17 | 30 | 106 | 3789 |
| austria | 1 | 17 | 26 | 16 | 1670 |
| egypt | 1 | 18 | 26 | 15 | 189 |
| thailand | 3 | 13 | 25 | 19 | 531 |
| japan | 3 | 12 | 24 | 82 | 3570 |
| ireland | 2 | 14 | 22 | 18 | 805 |
| romania | 1 | 17 | 22 | 19 | 464 |
| south korea | 3 | 11 | 22 | 71 | 3201 |
| singapore | 3 | 8 | 20 | 13 | 231 |
| new zealand | 3 | 8 | 18 | 19 | 628 |

**Supplementary Table 2.** Co-occurrence cluster analysis of top 30 high-output academic institutions

| label | cluster | weight<Links> | weight<Total link strength> | weight<Documents> | weight<Citations> |
| --- | --- | --- | --- | --- | --- |
| univ copenhagen | 3 | 45 | 45 | 81 | 16982 |
| univ helsinki | 2 | 30 | 30 | 47 | 10562 |
| univ gothenburg | 3 | 26 | 26 | 46 | 8898 |
| catholic univ louvain | 3 | 15 | 15 | 43 | 15725 |
| chinese acad sci | 1 | 23 | 23 | 38 | 1574 |
| shanghai jiao tong univ | 1 | 20 | 20 | 37 | 2256 |
| tabriz univ med sci | 5 | 5 | 5 | 35 | 1505 |
| zhejiang univ | 1 | 9 | 9 | 34 | 572 |
| china agr univ | 1 | 12 | 12 | 33 | 787 |
| univ turku | 2 | 27 | 27 | 31 | 3800 |
| univ florida | 2 | 16 | 16 | 30 | 2703 |
| sun yat sen univ | 1 | 14 | 14 | 29 | 556 |
| yale univ | 1 | 8 | 8 | 27 | 2434 |
| jiangnan univ | 1 | 13 | 13 | 27 | 782 |
| inserm | 3 | 17 | 17 | 26 | 4480 |
| univ toronto | 4 | 9 | 9 | 25 | 2062 |
| kashan univ med sci | 5 | 5 | 5 | 25 | 1314 |
| shandong univ | 1 | 13 | 13 | 25 | 424 |
| univ groningen | 3 | 21 | 21 | 24 | 4429 |
| univ amsterdam | 3 | 12 | 12 | 24 | 3454 |
| harvard med sch | 2 | 21 | 21 | 24 | 1279 |
| nanjing med univ | 1 | 15 | 15 | 24 | 318 |
| nanchang univ | 1 | 3 | 3 | 23 | 478 |
| beijing univ chinese med | 1 | 5 | 5 | 23 | 432 |
| nanjing univ chinese med | 1 | 2 | 2 | 23 | 344 |
| univ chinese acad sci | 1 | 12 | 12 | 22 | 871 |
| univ sao paulo | 4 | 8 | 8 | 22 | 833 |
| southern med univ | 1 | 11 | 11 | 22 | 524 |
| wageningen univ | 3 | 12 | 12 | 21 | 7082 |
| univ melbourne | 4 | 15 | 15 | 21 | 571 |

**Supplementary Table 3. Co-occurrence cluster analysis of top 30 cited journals**

| Journal | cluster | weight<Total link strength> | weight<Citations> |
| --- | --- | --- | --- |
| nature | 1 | 329205 | 6783 |
| diabetes | 2 | 253088 | 4903 |
| plos one | 1 | 221609 | 4591 |
| p natl acad sci usa | 1 | 191833 | 3541 |
| diabetologia | 2 | 142944 | 2894 |
| diabetes care | 2 | 127800 | 2779 |
| gut | 1 | 146921 | 2616 |
| science | 1 | 146173 | 2553 |
| brit j nutr | 3 | 101246 | 1967 |
| cell metab | 2 | 117410 | 1935 |
| sci rep-uk | 1 | 88771 | 1925 |
| am j clin nutr | 3 | 101187 | 1820 |
| cell | 1 | 104424 | 1812 |
| gastroenterology | 2 | 101968 | 1666 |
| nat med | 1 | 88812 | 1562 |
| nutrients | 3 | 71948 | 1506 |
| j nutr | 3 | 82701 | 1457 |
| j clin invest | 2 | 79968 | 1353 |
| appl environ microb | 1 | 57460 | 1271 |
| j biol chem | 2 | 71605 | 1146 |
| isme j | 1 | 54699 | 1090 |
| nat commun | 1 | 61764 | 1077 |
| front microbiol | 1 | 49595 | 1028 |
| new engl j med | 2 | 56543 | 1005 |
| cell host microbe | 1 | 57316 | 1001 |
| lancet | 2 | 53506 | 1001 |
| j agr food chem | 3 | 43993 | 982 |
| j immunol | 1 | 55846 | 965 |
| j clin endocr metab | 2 | 49631 | 838 |
| obesity | 2 | 46915 | 811 |

**Supplementary Table 4. Top ten WOS categories and occurrence frequency**

| **WOS categories** | **occurrence frequency** |
| --- | --- |
| ENDOCRINOLOGY & METABOLISM | 495 |
| NUTRITION & DIETETICS | 367 |
| FOOD SCIENCE & TECHNOLOGY | 244 |
| BIOCHEMISTRY & MOLECULAR BIOLOGY | 228 |
| MICROBIOLOGY | 225 |
| PHARMACOLOGY & PHARMACY | 197 |
| MULTIDISCIPLINARY SCIENCES | 175 |
| MEDICINE, RESEARCH & EXPERIMENTAL | 175 |
| IMMUNOLOGY | 141 |
| GASTROENTEROLOGY & HEPATOLOGY | 98 |

**Supplementary Table 5. New subject categories appearing more than five times**

| Name | Average year of occurrence  (Weighted average) | Number of occurrences |
| --- | --- | --- |
| BIOLOGY | 2020 | 28 |
| CHEMISTRY, MEDICINAL | 2019.58 | 45 |
| GERIATRICS & GERONTOLOGY | 2019.5 | 14 |
| TOXICOLOGY | 2019.43 | 22 |
| INTEGRATIVE & COMPLEMENTARY MEDICINE | 2019.39 | 43 |
| CHEMISTRY, MULTIDISCIPLINARY | 2019.38 | 58 |
| AGRICULTURE, MULTIDISCIPLINARY | 2019.22 | 27 |
| CHEMISTRY, APPLIED | 2019.2 | 56 |
| CHEMISTRY, MULTIDISCIPLINARY | 2019.19 | 58 |
| UROLOGY & NEPHROLOGY | 2019.14 | 9 |
| POLYMER SCIENCE | 2019.1 | 21 |
| PHARMACOLOGY & PHARMACY | 2019.08 | 197 |
| MEDICINE, GENERAL & INTERNAL | 2019 | 87 |

**Supplementary Table 6.** Co-occurrence cluster analysis of top 50 high-frequency academic keywords

| label | cluster | weight<Links> | weight<Total link strength> | weight<Occurrences> |
| --- | --- | --- | --- | --- |
| gut microbiota | 1 | 83 | 5217 | 1079 |
| obesity | 1 | 83 | 3787 | 684 |
| inflammation | 1 | 83 | 2842 | 515 |
| insulin-resistance | 1 | 83 | 2353 | 425 |
| intestinal microbiota | 1 | 83 | 2250 | 409 |
| diabetes | 1 | 80 | 1796 | 319 |
| type 2 diabetes | 1 | 79 | 1495 | 296 |
| probiotics | 3 | 83 | 1596 | 284 |
| microbiota | 2 | 83 | 1414 | 278 |
| chain fatty-acids | 1 | 82 | 1287 | 236 |
| diet | 2 | 83 | 1309 | 234 |
| metabolism | 2 | 82 | 1159 | 224 |
| glucose | 3 | 80 | 1226 | 218 |
| diet-induced obesity | 1 | 80 | 1095 | 187 |
| oxidative stress | 3 | 77 | 945 | 187 |
| metabolic syndrome | 1 | 80 | 974 | 172 |
| association | 2 | 80 | 955 | 170 |
| insulin sensitivity | 1 | 79 | 935 | 168 |
| bacteria | 2 | 82 | 910 | 167 |
| gut microbiome | 2 | 81 | 760 | 167 |
| mice | 1 | 80 | 844 | 159 |
| metagenome | 2 | 77 | 818 | 156 |
| mellitus | 3 | 76 | 729 | 149 |
| microbiome | 2 | 82 | 819 | 149 |
| risk | 2 | 75 | 655 | 146 |
| glucagon-like peptide-1 | 1 | 74 | 742 | 143 |
| double-blind | 3 | 77 | 774 | 140 |
| type 2 diabetes mellitus | 3 | 72 | 731 | 140 |
| insulin resistance | 1 | 81 | 836 | 137 |
| type 1 diabetes | 2 | 69 | 604 | 132 |
| metformin | 3 | 78 | 722 | 129 |
| health | 2 | 79 | 611 | 125 |
| disease | 2 | 79 | 596 | 124 |
| expression | 2 | 82 | 577 | 123 |
| adipose-tissue | 1 | 73 | 676 | 119 |
| high-fat diet | 1 | 80 | 674 | 118 |
| akkermansia-muciniphila | 1 | 76 | 633 | 112 |
| dysbiosis | 2 | 79 | 594 | 110 |
| mechanisms | 1 | 81 | 594 | 108 |
| impact | 2 | 78 | 590 | 107 |
| prebiotics | 3 | 73 | 622 | 107 |
| children | 2 | 75 | 508 | 97 |
| insulin | 3 | 78 | 454 | 97 |
| diabetes mellitus | 1 | 75 | 463 | 92 |
| weight-loss | 1 | 74 | 506 | 91 |
| butyrate | 2 | 78 | 502 | 90 |
| resistance | 1 | 73 | 428 | 83 |
| glycemic control | 3 | 70 | 444 | 82 |
| bariatric surgery | 1 | 66 | 425 | 79 |
| hyperglycemia | 3 | 73 | 422 | 79 |

**Supplementary Table 7. Top 15 highly cited articles in the “gut microbiota and diabetes”area**

| First Author | Title | Article type | Year of publication | Journal | Times cited | Country of corresponding author |
| --- | --- | --- | --- | --- | --- | --- |
| Cani PD.et al | Metabolic endotoxemia initiates obesity and insulin resistance | animal experiment | 2007 | Diabetes | 3608 | France |
| Qin, JJ.et al | A metagenome-wide association study of gut microbiota in type 2 diabetes | clinical research | 2012 | Nature | 3387 | China |
| Cani PD.et al | Changes in gut microbiota control metabolic endotoxemia-induced inflammation in high-fat diet-induced obesity and DM in mice | animal experiment | 2008 | Diabetes | 2816 | France |
| Tremaroli V.et al | Functional interactions between the gut microbiota and host metabolism | review | 2012 | Nature | 2476 | Sweden |
| Everard A.et al | Cross-talk between Akkermansia muciniphila and intestinal epithelium controls diet-induced obesity | animal experiment | 2013 | PNAS | 2200 | Belgium |
| Larsen N.et al | Gut microbiota in human adults with type 2 diabetes differs from non-diabetic adults | clinical research | 2010 | Plos One | 1616 | Denmark |
| Cani PD.et al | Changes in gut microbiota control inflammation in obese mice through a mechanism involving GLP-2-driven improvement of gut permeability | animal experiment | 2009 | Gut | 1584 | Belgium |
| Vrieze A.et al | Transfer of intestinal microbiota from lean donors increases insulin sensitivity in individuals with metabolic syndrome | clinical research | 2012 | Gastroenterology | 1573 | Netherlands |
| Karlsson FH.et al | Gut metagenome in European women with normal, impaired and diabetic glucose control | clinical research | 2013 | Nature | 1519 | Sweden |
| Vijay-Kumar M.et al | Metabolic syndrome and altered gut microbiota in mice lacking Toll-like receptor 5 | animal experiment | 2010 | Science | 1367 | USA |
| Li Wen.et al | Innate immunity and intestinal microbiota in the development of Type 1 diabetes | animal experiment | 2008 | Nature | 1343 | USA |
| Marcel Roberfroid.et al | Prebiotic effects: metabolic and health benefits | review | 2010 | British Journal of Nutrition | 1250 | Belgium |
| P D Cani.et al | Selective increases of bifidobacteria in gut microflora improve high-fat-diet-induced diabetes in mice through a mechanism associated with endotoxaemia | animal experiment | 2007 | Diabetologia | 1137 | Belgium |
| Gwen Tolhurst.et al | Short-chain fatty acids stimulate glucagon-like peptide-1 secretion via the G-protein-coupled receptor FFAR2 | animal experiment | 2012 | Diabetes | 1096 | UK |
| Janet G M Markle.et al | Sex differences in the gut microbiome drive hormone-dependent regulation of autoimmunity | animal experiment | 2013 | Science | 1041 | Canada |

**Supplementary Table 8. Top 15 articles with the highest burst strength in nearly 5 years**

| First Author | Title | Article type | Year of publication | Journal | Times cited | Country of corresponding author |
| --- | --- | --- | --- | --- | --- | --- |
| Hao Wu.et al | Metformin alters the gut microbiome of individuals with treatment-naive type 2 diabetes, contributing to the therapeutic effects of the drug | clinical research+animal experiment | 2017 | Nature Medicine | 666 | Sweden |
| Liping Zhao.et al | Gut bacteria selectively promoted by dietary fibers alleviate type 2 diabetes | clinical research+animal experiment | 2018 | Science | 820 | China |
| Forslund K.et al | Disentangling type 2 diabetes and metformin treatment signatures in the human gut microbiota | clinical research+animal experiment | 2015 | Nature | 1025 | Denmark |
| Aleksandar D Kostic.et al | The dynamics of the human infant gut microbiome in development and in progression toward type 1 diabetes | clinical research | 2015 | Cell Host Microbe | 581 | USA |
| Douglas J Morrison.et al | Formation of short chain fatty acids by the gut microbiota and their impact on human metabolism | review | 2016 | Gut Microbes | 1077 | Scotland |
| Tommi Vatanen.et al | The human gut microbiome in early-onset type 1 diabetes from the TEDDY study | clinical research | 2018 | Nature | 278 | USA |
| Ruud S Kootte.et al | Improvement of Insulin Sensitivity after Lean Donor Feces in Metabolic Syndrome Is Driven by Baseline Intestinal Microbiota Composition | clinical research | 2017 | Cell Metabolism | 403 | Sweden |
| [Eliana Mariño](https://pubmed.ncbi.nlm.nih.gov/?sort=date&term=Mari%C3%B1o+E&cauthor_id=28346408).et al | Gut microbial metabolites limit the frequency of autoimmune T cells and protect against type 1 diabetes | animal experiment | 2017 | Nature Immunology | 321 | Australia |
| Jacobo de la Cuesta-Zuluaga.et al | Metformin Is Associated With Higher Relative Abundance of Mucin-Degrading Akkermansia muciniphila and Several Short-Chain Fatty Acid-Producing Microbiota in the Gut | clinical research | 2017 | Diabetes Care | 299 | Colombia |
| Hubert Plovier.et al | A purified membrane protein from Akkermansia muciniphila or the pasteurized bacterium improves metabolism in obese and diabetic mice | animal experiment | 2017 | Nature Medicine | 784 | Belgium |
| Christoph A Thaiss.et al | Hyperglycemia drives intestinal barrier dysfunction and risk for enteric infection | animal experiment | 2018 | Science | 317 | Israel |
| Lulu Sun.et al | Gut microbiota and intestinal FXR mediate the clinical benefits of metformin | animal experiment | 2018 | Nature Medicine | 303 | China |
| Kristine H Allin.et al | Aberrant intestinal microbiota in individuals with prediabetes | clinical research+animal experiment | 2018 | Diabetologia | 150 | Denmark |
| Fernando F Anhê.et al | A polyphenol-rich cranberry extract protects from diet-induced obesity, insulin resistance and intestinal inflammation in association with increased Akkermansia spp. population in the gut microbiota of mice | animal experiment | 2015 | Gut | 637 | Canada |
| Annika Wahlström.et al | Intestinal Crosstalk between Bile Acids and Microbiota and Its Impact on Host Metabolism | review | 2016 | Cell Metabolism | 935 | Sweden |
